# Cucumber mosaic virus-induced gene silencing in banana

**DOI:** 10.1038/s41598-019-47962-3

**Published:** 2019-08-09

**Authors:** Yuh Tzean, Ming-Chi Lee, Hsiao-Hsuan Jan, Yi-Shu Chiu, Tsui-Chin Tu, Bo-Han Hou, Ho-Ming Chen, Chun-Nan Chou, Hsin-Hung Yeh

**Affiliations:** 1 Agricultural Biotechnology Research Center, Academia Sinica, No. 128, Section 2, Academia Road, Nankang District, Taipei, 11529 Taiwan; 2 Department of Plant Pathology and Microbiology, National Taiwan University, No. 1, Section 4, Roosevelt Road, Da’an District, Taipei, 10617 Taiwan

**Keywords:** Assay systems, RNAi

## Abstract

Banana (*Musa* spp.) is one of the world’s most important staple and cash crops. Despite accumulating genetic and transcriptomic data, low transformation efficiency in agronomically important *Musa* spp. render translational researches in banana difficult by using conventional knockout approaches. To develop tools for translational research in bananas, we developed a virus induced-gene silencing (VIGS) system based on a banana-infecting cucumber mosaic virus (CMV) isolate, CMV 20. CMV 20 genomic RNA 1, 2, and 3, were separately cloned in *Agrobacterium* pJL89 binary vectors, and a cloning site was introduced on RNA 2 immediately after the 2a open reading frame to insert the gene targeted for silencing. An efficient *Agrobacterium* inoculation method was developed for banana, which enabled the CMV 20 VIGS vector infection rate to reach 95% in our experiments. CMV 20-based silencing of *Musa acuminata* cv. Cavendish (AAA group) *glutamate 1-semialdehyde aminotransferase* (*MaGSA*) produced a typical chlorotic phenotype and silencing of *M. acuminata phytoene desaturase* (*MaPDS*) produced a photobleachnig phenotype. We show this approach efficiently reduced *GSA* and *PDS* transcripts to 10% and 18% of the control, respectively. The high infection rate and extended silencing of this VIGS system will provide an invaluable tool to accelerate functional genomic studies in banana.

## Introduction

Bananas belong to the family Musaceae and represent one of the world’s most important cash and staple crops^1^. This perennial crop is cultivated throughout the tropics and sub-tropics, and over 400 million people in developing regions are dependent on bananas as a vital food nutrient. In 2017, worldwide production of bananas was estimated to be over 113 million tons^2^. The vast majority of cultivated bananas are triploid, and are derived from inter- and intra-specific crosses between two diploid wild species, *Musa acuminata* (A genome) and *M*. *balbisiana* (B genome)^3^. Currently, commercialized banana is dominated by the Cavendish (AAA) cultivar group^4^.

The genome of *M. acuminata* ssp. *malaccensis*, a progenitor of the current cultivated banana, was the first *Musa* genome sequenced^5^, followed by a draft release of the genome of another banana progenitor, *M. balbisiana*^6^, and a close relative, *M. itinerans*^7^. In addition, a number of gene expression profiles of different banana cultivars, tissues, and conditions have also been investigated^8–14^. Despite the success of banana genomic and transcriptomic sequencing projects, reverse genetic approaches for identifying and characterizing gene functions in banana remain a challenge.

To date, characterizing target gene(s) of interest in banana has mainly relied on transgenic banana plants overexpressing the exogenous target gene^15–17^. More recently, a novel transgenic approach based on clustered regularly interspaced short palindromic repeats (CRISPR)/CRISPR-associated protein 9 (Cas9) are being reported for plants, and the use of CRISPR-Cas9 has been reported in banana cv. Rasthali^18^. However, low transformation efficiency and the time-exhaustive regeneration processes involved in this transgenic approach are disadvantageous in functional characterization of one or several candidate genes using CRISPR-Cas9 in banana. Moreover, polypoidy especially in the triploid Cavendish banana presents additional challenges for genetic knockout analysis^19^, as knockout of the target gene in all sets of chromosomes would be needed for loss-of-function analysis.

The availability of genomic and transcriptomic data have provided the basis for reverse genetic studies^5–9,13,20–22^. Virus-induced gene silencing (VIGS) has been used extensively as a tool for reverse genetic study in a wide range of plant species to characterize gene functions or for high throughput phenotypic screening of genes^23–34^. Instead of complete knockout of a gene, the VIGS system knocks down a gene at the RNA level thereby overcoming polyploidy concerns^30^. Such a system offers an attractively rapid method for loss-of-function assay^23,35^ in banana by circumventing the laborious transformation method. The ease of the VIGS system has led to the development of several VIGS vectors being used for gene function analysis or for high throughput phenotypic screening of genes in dicotyledonous plants. However, less vectors are available for studies in monocotyledonous plants^26,29,32,36–39^.

Cucumber mosaic virus (CMV) is known to infect a wide host range of more than 1200 species in over 100 plant families, including banana^37–39^. CMV is the type member of the genus *Cucumovirus* and consists of three positive sense single-stranded genomic RNAs (RNA1, 2, and 3) that expresses five proteins (see Supplementary Fig. S1)^40–42^. RNA1 and RNA2 encode the 1a and 2a proteins, respectively, involved in viral genome replication^43^. Additionally, RNA2 contains the 2b gene, which encodes the suppressor of RNA silencing^41,44^. The 2b has been reported to suppress RNA silencing through binding to short-interfering RNAs or double-stranded RNAs^45,46^, and impairing Argonaute 1 and 4 protein activities by direct interaction^47–52^. Previous studies indicate that without functional 2b gene, CMV systemically infects plants with mild symptoms^53,54^. RNA3 encodes the 3a movement protein and coat protein (CP). The CMV CP is a multifunctional factor that has roles in viral packaging as well as in systemic movement^42^. CMV has been successfully deployed as a VIGS in dicotyledonous plants such as *Nicotiana benthamiana*^55^, *Glycine max*^56^, *Antirrhinum majus*^57^, *Solanum lycopersicum*, and *Capsicum annuum*^58^, as well as monocotyledonous maize^59^; whether CMV can induce silencing in the monocotyledonous banana remains unknown.

It has been demonstrated that the successful development of VIGS vector largely depends on the virus strain^26,55,59^. Previously, we reported isolation of number of CMV isolates from bananas grown in the field^60^. Subsequently, by selecting a naturally banana-infecting CMV isolate and improving plant infection rates we successfully established a robust VIGS-based system in bananas. The developed tool will greatly expedite gene validation and selection prior to use in transgenic crops.

## Results

### Development of CMV VIGS vector

Initially, to analyze the suitability of the cucumber mosaic virus 20 (CMV 20)^60^ previously isolated from banana to induce gene silencing, we constructed cDNA infectious clones of CMV 20 RNAs 1, 2, and 3 (see Supplementary Fig. S2). The infectious clones contained T_3_ promoter immediately upstream of the full-length cDNA of CMV 20 RNAs 1, 2, and 3 (see Supplementary Fig. S2). We designated the CMV 20 RNA1 as pCMV20-R1, CMV 20 RNA2 as pCMV20-R2, and CMV 20 RNA3 as pCMV20-R3 (see Supplementary Fig. S2). No modification to full-length cDNA of CMV 20 RNAs 1 and 3 were made, whereas an AfeI restriction enzyme site was introduced in a position similar to that previously described^55^, which is immediately downstream of 2a and in the 2b ORF of CMV to generate pCMV20-R2E (see Supplementary Fig. S2). The AfeI site served as a cloning site for our target gene, and disrupts CMV 20 when a target gene fragment is inserted. VIGS vector construction is detailed in the Materials and Methods section.

### CMV 20 induces gene silencing in *N. benthamiana* by agroinfection

**Figure 1:**
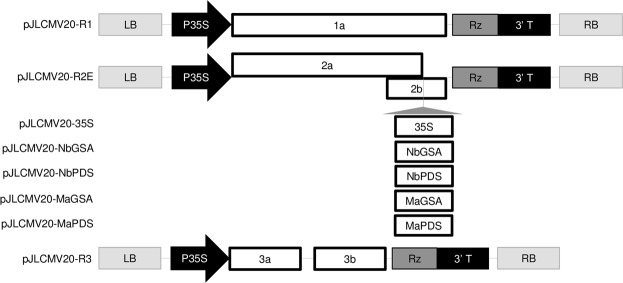
Schematic representation of CMV 20 VIGS vectors. pJLCMV20-R1, pJLCMV20-R2E, and pJLCMV20-R3 were generated by cloning CMV 20 RNA1, RNA2E (RNA2 containing AfeI restriction enzyme site), and RNA3 to binary vector pJL89, respectively. T-DNA left border (LB) and right border (RB) of the binary vector (pJL89) are indicated in light grey. Open rectangles represent open reading frames (ORF) encoded by CMV genomic RNAs or of the inserted DNA fragments (35S, NbGSA, NbPDS, MaGSA, and MaPDS). 35S promoter (35S) is indicated with black arrow immediately adjacent to the most 5′ end of CMV 20 ORF, and hepatitis delta virus ribozyme (Rz) to the 3′ end. Schematic depiction of AfeI cloning site is indicated with a grey line and triangle.

As we were unable to inoculate *in vitro* transcripts of CMV 20 vector on banana, we sought alternative methods using agroinfection. We cloned CMV 20 genomic RNA 1, 2 and 3 from pCMV20-R1, -R2E, and -R3 to binary vector pJL89^61^ to generate pJLCMV20-R1, pJLCMV20-R2E, and pJLCMV20-R3 (Fig. 1a). We inserted a DNA fragment of *CaMV* 35S promoter (35S) in pJLCMV20-R2E AfeI cloning site, which disrupted the CMV 20 2b gene, and designated it as pJLCMV20-35S (Fig. 1a). 35S promoter is a foreign DNA, which is not endogenously found in *N. benthamiana* or banana. Thus, pJLCMV20-35S was subsequently used as a control vector for pJLCMV20-R2E carrying host endogenous target gene in VIGS assay. DNA fragment of NbGSA and *N*. *benthamiana phytoene desaturase* gene (NbPDS) were inserted to pJLCMV20-R2E to generate pJLCMV20-NbGSA and pJLCMV20-NbPDS, respectively (Fig. 1a).

**Figure 2:**
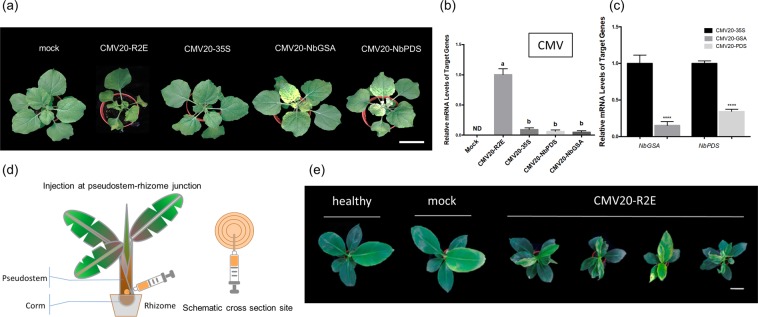
Agro-inoculation of *Nicotiana benthamiana* and banana with CMV 20 VIGS vectors. (**a**) Phenotypes of *N. benthamiana* inoculated with *Agrobacterium* containing pJL89 (mock), or *Agrobacterium* mixture carrying pJLCMV20-R1, pJLCMV20-R3 plus pJLCMV20-R2E (CMV20-R2E), pJLCMV20-35S (CMV20-35S), pJLCMV20-NbGSA (CMV20-NbGSA), or pJLCMV20-NbPDS (CMV20-NbPDS) at 8 dpi. Bar = 5 cm. (**b**) Quantification of the mRNA levels of CMV 20 coat protein in *N. benthamiana* inoculated with mock, CMV20-R2E, CMV20-35S, CMV20-NbGSA, and CMV20-NbPDS. Data represent mean ± SD, n = 5 biological replicates. Different letters indicate statistically significant differences analyzed by one-way analysis of variance (ANOVA) Tukey’s test (*P* < 0.05). (**c**) Quantification of the mRNA levels of *NbPDS*, and *NbGSA* of *N. benthamiana* analyzed by quantitative RT-PCR, normalized relative to the level of actin at 8 dpi. Data represent mean ± SD; n = 5 biological replicates; ND, not detectable. ****P < 0.001, Student’s t test compared to CMV20-35S agro-inoculum inoculated plants. (**d**) Schematic representation of *Agrobacterium*-assisted CMV 20 inoculation method by injecting *Agrobacterium* mixture carrying CMV 20 into bananas at the pseudostem-rhizome junction. (**e**) Phenotypes of healthy, mock inoculated, or CMV20-R2E agro-infected banana plants. Scale bar = 10 cm.

CMV20-R2E agroinfection (a mixture of *Agrobacterium* carrying pJLCMV20-R1, pJLCMV20-R3, and pJLCMV20-R2E), CMV20-35S agroinfection (a mixture of *Agrobacterium* carrying pJLCMV20-R1, pJLCMV20-R3, and pJLCMV20-35S), CMV20-NbGSA agroinfection (a mixture of *Agrobacterium* carrying pJLCMV20-R1, pJLCMV20-R3, and pJLCMV20-NbGSA), and CMV20-NbPDS agroinfection (a mixture of *Agrobacterium* carrying pJLCMV20-R1, pJLCMV20-R3, and pJLCMV20-NbPDS) were infiltrated with a needleless syringe onto *N*. *benthamiana* leaves and assayed for infectivity and silencing efficacy. Phenotypically, CMV20-R2E infected *N. benthamiana* plants scored an average disease severity level of 3, whereas CMV20-35S agro-inoculated plants exhibited mild (disease severity level of 1) to asymptomatic phenotype (Fig. 2a, see Supplementary Fig. S3). In addition, visible chlorotic and photobleaching phenotype was observed in all plants agroinfected with CMV20-NbGSA or of CMV20-NbPDS at 6–8 dpi, respectively (Fig. 2a).

Total RNA extracted from mock, CMV20-R2E, CMV20-35S, CMV20-NbGSA, and CMV20-NbPDS agro-infiltrated plants at 8 dpi were used for detection of CMV by real- time qRT-PCR. The infection of CMV was confirmed in all CMV20-R2E, CMV20-35S, CMV20-NbGSA, and CMV20-NbPDS agro-infiltrated plants in our three independent experiments (Fig. 2b). In addition, significant reduction of *GSA* and *PDS* mRNA was observed in plants with CMV20-NbGSA and CMV20-NbPDS agroinfection, respectively, compared to CMV20-35S agroinfected plants (Fig. 2c).

### Development of efficient CMV 20 vector inoculation methods on banana by pseudostem-rhizome junction injection of *Agrobacterium*

Despite successfully agro-infiltrating CMV 20 vectors in *N. benthamiana*, we were not able to transmit CMV 20 vectors in banana using a regular agro-infiltration method. Therefore, we optimized an agroinfection method for banana. We used inoculation buffer that has been reported to enable high inoculation efficiency^62,63^, and used needled injection instead of needleless infiltration of *Agrobacterium*-mixture to minimize the wounding as we previously demonstrated on orchids^32,64^. In addition, we tested agro-injection or CMV 20 vector on different banana tissues (Fig. 2d). In our repeated sets of experiments, 95% (41 of 43 plants) of bananas inoculated with CMV20-R2E at the pseudostem-rhizome junction showed severe viral symptoms, similar to the slender leaves previously described^60^, starting at 25 dpi to 36 dpi (Fig. 2e). In addition to symptom observation, qRT-PCR was used to detect CMV in ten randomly selected plants that had been inoculated with CMV20-R2E, which confirmed consistent CMV infection.

### Silencing of the banana *GSA* and *PDS* genes by CMV 20 vectors

Building on the success of our inoculation system, we next tested whether CMV 20 vector could be used to induce gene silencing in bananas. A 205-bp *Musa* GSA (MaGSA) and a 204-bp *Musa* PDS (MaPDS) DNA fragment were cloned into pJLCMV20-R2E to generate pJLCMV20-MaGSA and pJLCMV20-MaPDS, respectively (Fig. 1). CMV20-R2E, CMV20-35S, CMV20-MaGSA (mixture of *Agrobacterium* carrying pJLCMV20-R1, pJLCMV20-R3, and pJLCMV20-MaGSA), or CMV20-MaPDS (mixture of *Agrobacterium* carrying pJLCMV20-R1, pJLCMV20-R3, and pJLCMV20-MaPDS) were agroinfected into banana plants.

**Figure 3:**
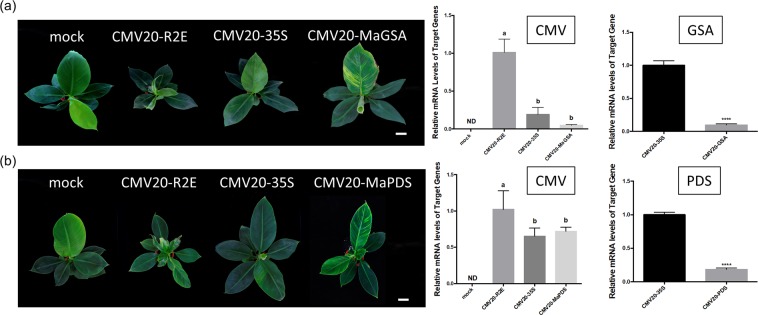
CMV20-based VIGS of *glutamate 1-semialdehyde aminotransferease* (*MaGSA*) or *phytoene desaturase* (*MaPDS*) in banana. (**a**) Phenotype of banana inoculated with *Agrobacterium* containing pJL89 (mock), or *Agrobacterium* mixture carrying pJLCMV20-R1, pJLCMV20-R3 plus pJLCMV20-R2E (CMV20-R2E), pJLCMV20-35S (CMV20-35S), or pJLCMV20-MaGSA (CMV20-MaGSA) at 30 dpi. Bar = 5 cm. Quantification of the mRNA levels of CMV 20 coat protein and *MaGSA* was analyzed by quantitative RT-PCR, normalized relative to the level of actin. (**b**) Phenotypes of banana inoculated with *Agrobacterium* containing pJL89 (mock), CMV20-R2E, CMV20-35S, or pJLCMV20-MaPDS (CMV20-MaPDS) at 30 dpi. Bar = 5 cm. Quantification of the mRNA levels of CMV20 coat protein and *MaPDS* was analyzed by quantitative RT-PCR, normalized relative to the level of actin. Quantitative RT-PCR data represent mean ± SD; 3 representative technical repeats; ND, not detectable. Different letters indicate statistically significant differences analyzed by one-way analysis of variance (ANOVA) Tukey’s test (*P* < 0.05).). ****P < 0.001, Student’s t test compared to CMV20-35S agro-inoculum inoculated plants.

In our experiments, banana inoculated at 25 °C for 2 weeks and then shifted to 28 °C showed *GSA* silencing phenotype that could be observed on CMV20-MaGSA agroinfected banana starting at 30 dpi (Fig. 3a, Table 1). In three repeated sets of experiments, the chlorotic phenotype was observed in at least 70% and up to 80% of CMV20-MaGSA agroinfected bananas (Table 1), and the photobleaching phenotype was observed in at least 60% and up to 85% of CMV20-MaPDS agroinfected bananas (Experiments 2 and 3, Table 1). The majority of the CMV20-35S, CMV20-MaGSA, or CMV20-MaPDS agroinfected bananas were asymptomatic or mildly symptomatic, and severe symptoms were rarely observed (1 out of 28 CMV20-35S agroinfected bananas, 1 out of 28 CMV20-MaGSA agroinfected bananas, and 1 out of 23 CMV20-MaPDS agroinfected bananas) (Experiments 1–3, Table 1). In addition to CMV20-R2E, real-time qRT-PCR also revealed that the infection rate reached 92%, 100%, and 100% for bananas with CMV20-35S agroinfection, CMV20-MaGSA agroinfection, and CMV20-MaPDS agroinfection, respectively (Experiment 3, Table 1).

**Table 1:** Ratio of infectivity and morphological change in inoculated bananas.

Inoculum^†^	Experiment 1		Experiment 2		Experiment 3		
	Severe Symptom^‡^	Silencing Phenotype^§^	Severe Symptom	Silencing Phenotype	Severe Symptom	Silencing Phenotype	Infection Rate^||^
mock	0/5	0/5	0/5	0/5	0/5	0/5	0/5
CMV20-R2E	5/5	0/5	5/5	0/5	2/2	0/2	2/2
CMV20-35S	0/5	0/5	0/10	0/10	1/13	0/13	12/13
CMV20-MaGSA	1/5	4/5	0/10	7/10	0/13	10/13	13/13
CMV20-MaPDS	—	—	1/10	6/10	0/13	11/13	13/13

^†^Mock, CMV20-R2E, CMV20-35S, CMV20-MaGSA, or CMV20-MaPDS agroinfected plants.

^‡^Ratio of plants showing severe symptom/total number of specified inoculated plants.

^§^Ratio of plants showing silencing phenotypes/total number of tested plants.

^‖^Ratio of infected plants/total number of specified inoculated plants. Infection was determined based on qRT-PCR detection.

-No plants tested.

The silencing phenotypes that could be observed starting around 30 dpi (Fig. 3a,b) could also be observed in newly developed leaves up to 63 dpi. In addition, the results of qRT-PCR revealed that the RNA level of *MaGSA* in CMV20-MaGSA agroinfected bananas and *MaPDS* in CMV20-MaPDS agroinfected bananas was significantly reduced by 90% and 82% of the CMV20-35S agroinfected bananas (Fig. 3a,b), respectively, and corresponded to the phenotype observed. Taken together, these data showed that the currently developed CMV 20-based VIGS system could be effectively used to silence genes of interest in banana plants.

### CMV 20-vectors induce generation of siRNA

**Figure 4:**
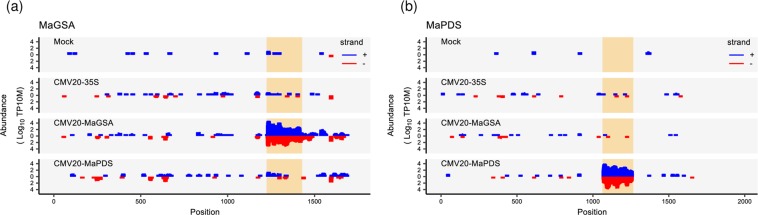
Coverage plots of 21–24 nt small RNAs on sense and antisense strands of MaGSA (**a**) or MaPDS (**b**). TP10M: tags per 10 million. The regions corresponding to gene fragments inserted into the VIGS vector are highlighted in orange.

To further confirm that reduced expression of *GSA* and *PDS* RNA was due to RNA interference caused by VIGS, small RNA-seq was conducted using mock, CMV20-35S, CMV20-MaGSA, and CMV20-MaPDS agro-inoculum inoculated plants. Short sense and antisense reads of 21–24 nt were mapped to the cDNA sequences of MaGSA and MaPDS (Fig. 4). Although short sense and antisense reads that mapped to either MaGSA or MaPDS can be identified in all plant groups (mock, CMV20-35S, CMV20-MaGSA, and CMV20-MaPDS agro-inoculum inoculated plants), the relative abundance of sense and antisense reads of MaGSA and MaPDS was significantly higher in CMV20-MaGSA and CMV20-MaPDS bananas compared to control plants (mock and CMV20-35S plants), respectively. In CMV20-MaGSA agroinfected bananas, the small RNA (21–24 nt) abundance mapped to MaGSA was 1052 fold higher than the mock and 446 fold higher than the CMV20-35S agroinfected bananas (see Supplementary Table S1). In CMV20-MaPDS agroinfected bananas, the small RNA (21–24 nt) abundance mapped to MaPDS was 1195 fold higher compared to mock and 451 fold higher compared to CMV20-35S agroinfected bananas (see Supplementary Table S1). These results indicate the target specificity of the established VIGS using CMV 20 as viral vector.

## Discussion

Here, we report the development of a robust and efficient CMV-VIGS system for bananas and demonstrated that this approach enabled efficient silencing of genes using *PDS* and *GSA*. The developed CMV 20 vector allows silencing of targeted genes of interest with high infection rate and mild disease symptoms. The success of our VIGS system in banana may be partly attributed to using a naturally banana infecting CMV isolate for the construction of banana VIGS system, as well as overcoming the difficulties of CMV 20 inoculation on banana using a pseudostem-rhizome junction needled agroinjection system (Fig. 2d).

In our study, we tested a number of methods to inoculate CMV 20 empty vector or CMV 20 vector with a targeted silence gene insert. While agro-inoculation systems have been widely adopted for virus delivery into plants^65–67^, inoculation of plant viruses with an agrobacterium-assisted system on *Musa* plants has not been successfully established prior to this study. Compared to inoculating CMV 20 on *N*. *benthamiana*, the thicker banana leaves, latex, and other inhibitory substances present in bananas add to the challenge of establishing an efficient viral inoculation system^68,69^. Of several inoculation methods, we were able to obtain a desirable infection rate (Table 1) by replacing regular needleless agro-infiltration methods with a syringe and fine needle for inoculum injection. Such an approach may allow minimal wounding on banana, and injecting an *Agrobacterium* mixture carrying CMV 20 to the center of banana pseudostem-rhizome juncture, likely allowed virus infection to take place on the meristem or yet-to-emerge leaves before the true banana leaf is developed. Interestingly, it has been previously reported that 2b protein-defective mutant is unable to invade the shoot apical meristem^70^. This may partly explain why CMV 20 accumulation is significantly higher in CMV20-R2E agroinfected plants compared to CMV20-GSA or CMV20-PDS.

The *GSA* and *PDS* genes are commonly used as phenotypic markers for VIGS vector development in many plant species^59^. Using CMV 20-based VIGS vector, silencing *MaGSA* and *MaPDS* in banana resulted in a chlorotic or photobleaching phenotype, respectively, that was observed starting 30 dpi and persisted until 63 dpi. We speculate that the meristem tissue is vulnerable to transformation of agrobacterium, and serves as the initial infection site for CMV. As no vascular system in the meristem is available for virus movement, virus infection is confined to the meristem, and VIGS phenotype is only visualized with the development of the leaves. Moreover, the visual phenotype was observed in 77% or 85% of the infected bananas inoculated with CMV20-MaGSA agro-inoculum or CMV20-MaPDS agro-inoculum, respectively (Experiment 3, Table 1). We found initial temperature set at 25 °C for two weeks followed by constant 28 °C provided the best VIGS results compared to constant temperature maintained at 28 °C. It has been reported that lower temperature during inoculation may increase the transformation efficiency of *Agrobacterium*^71^. The temperature control allowed us to achieve a balance for plant growth and VIGS efficacy.

Small RNA sequencing from mock, CMV20-35S agro-inoculum, CMV20-MaGSA agro-inoculum, and CMV20-MaPDS agro-inoculum inoculated banana revealed that higher levels of small RNAs were mapped to the targeted gene and corresponded to the reduction in the mRNA transcript level of the targeted gene, and this higher level of *MaGSA* and *MaPDS* small RNAs are found in both the sense and anti-sense orientation indicating that double stranded small RNA is formed and induced post-transcriptional silencing response in this VIGS system.

In this study, the CMV 20-based VIGS vector developed effectively silenced endogenous genes in both *N*. *benthamiana* and banana, demonstrating that CMV 20 is able to induce silencing in both dicots and monocots, respectively. We demonstrated that the combination of the developed CMV 20 vector and inoculation strategy enabled silencing of the targeted gene of interest in banana with high infection rate and silencing efficacy. Although VIGS may be limited by the duration of silencing or inherently having to observe plant phenotype under virus infection, the availability of the system developed here may enable previously unavailable rapid functional analysis of genes in banana and expedite a more complete understanding of different biological questions in this important staple and cash crop.

## Materials and Methods

### Plant material and growth conditions

*Nicotiana benthamiana* was grown in pots at 25 °C, in insect-proof growth chamber under 16 h light/8 h dark cycle with 60 μmol m^−2^ s^−1^ illumination. ‘Tai-Chiao No. 7’ (Cavendish, AAA subgroup) plantlets were purchased from Taiwan Banana Research Institute (Pingtung, Taiwan). Unless otherwise indicated, banana plants in jars were first placed in growth chamber for seven days for acclimation prior to potting in sterilized soil containing 6 parts peat, 1 part perlite, and 1 part vermiculite, and grown in insect-proof growth chamber at 28 °C under 16 h light/8 h dark cycle with 60 μmol m^−2^ s^−1^ illumination. For VIGS experiments, unless otherwise specified, inoculated banana plantlets were placed at 25 °C under 16 h light/8 h dark cycle with 60 μmol m^−2^ s^−1^ illumination for 2 weeks and transferred to 28 °C of the same illumination.

### Construction of infectious CMV20-derived vectors

All primers used are listed in Supplementary Table S2. The full-length cucumber mosaic virus (CMV20) cDNA of RNAs 1, 2, and 3 (NCBI accession number: MH709142-MH709144) were first cloned into pGEM-T vectors (Promega, Madison, WI, USA) and designated pCMV20-R1, pCMV20-R2, and pCMV20-R3. Total RNA extracted from CMV20 infected *N. benthamiana* using TRIzol reagent (Thermo Fisher Scientific, Waltham, MA, USA) according to the manufacturer’s instructions, was used as template for synthesis of first strand cDNA of CMV 20 RNA1 by use of CMV 3′ primer and M-MLV Reverse Transcriptase (Promega). Two overlapping CMV20 RNA 1 fragments were PCR amplified using primer pairs R125 (with T_3_ promoter)/YR1 1646RE and YR1 1584FR/CMV3′. The two amplified fragments were cloned into pGEM-T (Promega). The two plasmids were digested with EcoRV and NotI and separated on 1% agarose gel. The 4.6 and 1.7 kb fragments were gel purified and ligated to generate pCMV20-R1.

The construction of pCMV20-R2 is similar to construction of pCMV20-R1 except primer pairs R125 (with T_3_ promoter)/YR2 1720RE and YR2 1536FR/CMV3′ were used instead to amplify two overlapping CMV fragments for cloning to pGEM-T (Promega), and both cloned plasmids were digested with XhoI and SpeI then, separated on 1% agarose gel. The 4.5 kb and 1.5 kb fragments were gel purified and ligated to generate pCMV20-R2.

For construction of pCMV20-R3, primer pair RNA35′20-2 (with T_3_ promoter)/CMV 3′ were used to amplify full-length CMV 20 RNA3. The amplified fragment was cloned into pGEM-T easy (Promega) to generate pCMV20-R3.

An AfeI restriction enzyme site was added on the 2b ORF to generate a gene insertion site similar to the method previously described^55^. Two fragments were amplified by PCR with pCMV20-R2 used as template and primer pairs CMV2-2225F/CMV2-2663AfeIR and CMV2-2663AfeIF/CMV2-294R. The amplified fragments were gel extracted and mixed as templates for overlapping extension PCR reaction by use of primer pairs CMV2-2225F/CMV2-2949R. The amplified fragments were gel extracted and digested with SfoI and AvrII. The digested fragments were ligated into pCMV20-R2 that had been digested with SfoI and AvrII to generate pCMV20-R2E.

To generate pJLCMV20-R1, pCMV20-R1 was used as template and was amplified by PCR with primer pairs CMV20-R1R2-F/CMV20-R1-R. The amplified fragment was ligated to pJL89^61^ digested with StuI and SmaI. The construction of pJLCMV20-R2E and pJLCMV20-R3 followed a similar method to the construction of pJLCMV20-R1 except pCMV20-R2E and pCMV20-R3 were used as a template, and primer pairs CMV20-R1R2-F/CMV20-R2-R and CMV20-R3-F/CMV20-R3-R were used to amplify the CMV20-R2E and CMV20-R3 fragments in the PCR reaction and ligated to pJL89^61^.

### Construction of CMV20 gene-silencing vectors

Total RNA extracted from *N. benthamiana* using TRIzol reagent (Thermo Fisher Scientific) according to the manufacturer’s instructions, was used to synthesize cDNA with PrimeScript RT-PCR Kit (TaKaRa Bio Inc., Otsu, Shiga, Japan). The gene fragment of *NbPDS* (NCBI accession number: EU165355.1) was amplified by using the synthesized cDNA and primer pair NbPDS-F/NbPDS-R. The amplified fragment was cloned into AfeI-digested pJLCMV20-R2E to generate pJLCMV20-NbPDS. The PCR amplification of NbGSA (Sol Genomic Network sequence ID: Niben101Scf06382g00006.1) fragment is similar to amplification of NbPDS fragment except NbGSA-F/NbGSA-R primer pair was used instead. The amplified NbGSA was cloned into AfeI digested pJLCMV20-R2E to generate pJLCMV20-NbGSA. 35S DNA fragment was PCR amplified with template pCymMV-Gateway vector (Lu *et al*., 2012) and primer pair 35S-F/35S-R, and cloned into AfeI-digested pJLCMV20-R2E to generate pJLCMV20-35S.

The construction of pJLCMV20-MaPDS and pJLCMV20-MaGSA was similar to that described above for pJLCMV20-NbPDS and pJLCMV20-NbGSA except total RNA was extracted from banana using PureLink Plant RNA Reagent (Thermo Fisher Scientific) and *MaPDS* (NCBI accession number: XM_018821595.1); and *MaGSA* (NCBI accession number: XM_009405501.2) fragments were amplified by primers MusaPDS-F/R or MusaGSA-F/R and cloned into the AfeI site of the pJLCMV20-R2E, which yielded plasmids pJLCMV20-MaPDS and pJLCMV20-MaGSA, respectively.

### Agroinfection of *N. benthamiana* and banana

All T-DNA clones were separately introduced into *A. tumefaciens* EHA105 by electroporation using a Gene Pulser (Bio-Rad Laboratories, Hercules, CA, USA). For *N*. *benthamiana*, the vectors pJLCMV20-R1, pJLCMV20-R2E, pJLCMV20-R3, pJLCMV20-35S, pJLCMV20-NbPDS, pJLCMV20-NbGSA were transformed individually into *A. tumefaciens* strain EHA105. Preparation of *Agrobacterium* cultures harboring pJLCMV20-R1, pJLCMV20-R2E, pJLCMV20-R3, or pJLCMV20-R2E derivatives carrying the specified gene inserts were grown and prepared as described previously^62^. Selective medium kanamycin of 50 mg in 1 L of Yeast Extract Peptone (YEP) liquid medium was used. The cell mixtures with 1:1:1 ratio between pJLCMV20-R1, pJLCMV20-R2E, pJLCMV20-R3, or pJLCMV20-R2E derivatives carrying the specified gene inserts (i.e. 35S, *NbPDS*, *NbGSA*) were infiltrated into true leaves of *N*. *benthamiana* plants. Mock plants were infiltrated with infiltration solution containing *A. tumefaciens* strain EHA105. Systemic leaves were collected at 8 days post-inoculation (dpi) for further analysis.

For bananas, preparation of the inoculum was similar to that previously described. For inoculation of banana, aliquots of 400 μl of *Agrobacterium* mixture was injected into pseudostem-rhizome junction of banana plants (1-month after transplanting tissue culture plantlets to soil pots) using a 0.5-ml sterile syringe.

### RT-PCR and qRT-PCR analysis

For *N*. *benthamiana*, total RNAs were extracted using TRIzol Reagent (Thermo Fisher Scientific) from 1 mg of leaf sample, and treated with Turbo DNAse (Thermo Fisher Scientific) to remove genomic DNA. cDNA for qRT-PCR was synthesized from 1 *μg* of DNA-free RNA and oligo (dT) using PrimeScript RT Reagent Kit (Takara Bio), following the manufacturer’s instructions. For banana samples, total RNAs were extracted using Purelink Plant Reagent (Thermo Fisher Scientific), and the synthesis of cDNA followed the same instructions as *N*. *benthamiana*.

For detection and quantification of CMV20 in both *N. benthamiana* and banana, primers pairs CMV20CP-F/R that targeted CMV20 coat protein were used. All qRT-PCR was carried out using SYBR Premix EX Taq II Kit (Takara Bio) with ABI Prism 7500 sequence detection system (Applied Biosystems). Actin of *N. benthamiana* or banana was used as a control to calculate the relative CMV20 level.

For qRT-PCR assay of silencing experiments, gene-specific primers were designed to prime outside the region targeted for silencing. For qRT-PCR of *N. benthamiana* PDS or GSA, primer pairs NbPDS-qF/qR or NbGSA-qF/qR were used, respectively. *N. benthamiana actin* (NCBI accession number: JQ256516.1) was used as an internal control using primer pairs NbActin-qF/qR. For banana PDS or GSA, primer pairs MusaPDS2-qF/qR or MusaGSA-qF/qR were used, respectively. Banana *actin* (NCBI accession number: XM_009412781.2) was used as in internal control using primer pairs MusaActin-qF/qR. RNA derived from mock- or 35S control vector-inoculated plants, as specified in the figures, was set as 1 for relative quantitative analysis.

### Small RNA sequencing and small RNA-Seq raw read processing

Total RNA of mock, CMV20-35S, CMV20-MaGSA, and CMV20-MaPDS were extracted from banana using PureLink Plant Reagent (Thermo Fisher Scientific) and sent underwent small RNA sequencing conducted by Biotools (Taipei, Taiwan). In brief, the small RNA was size excised from agarose gel and used as input material for a small RNA library construction using NEBNext Multiplex Small RNA Library Prep Set for Illumina (New England Biolabs, Ipswich, MA, USA) according to the manufacturer’s protocol. The libraries were pooled and sequenced on NextSeq 500 (Illumina, San Diego, CA, USA), and the sequenced data were uploaded into the NCBI Sequence Read Archive (Accession number: SRP159135). For raw read processing, the FastX-Toolkit v.0.0.14 was used to trim the adapter and for quality control. The trimmed reads of 18–28 nt were quality-filtered with patterns [-q 30 -p 90]. Next, the trimmed reads were mapped to the banana genome and cDNA sequences retrieved from the banana genome hub (*Musa acuminata* DH-Pahang version 2; banana-genome-hub.southgreen.fr) by BOWTIE 1.2.1.1 with 1 mismatch. The reads with >20 genomic hits or mapped to the mitochondria genome, chloroplast genome from GenBank (HF677508.1), non-coding RNA from Rfam 13.0, such as ribosomal RNAs, transfer RNAs, small nuclear RNAs and small nucleolar RNAs, were discarded. Reads derived from the transcript of MaPDS and MaGSA sequences were identified using BOWTIE 1.2.1.1 with 1 mismatch.

## Supplementary information

Supplementary Figures and Tables
