# Supplementary material for: Cucumber mosaic virus-induced gene silencing in banana
Source: Sci Rep. 2019 Aug 9;9:11553. doi: 10.1038/s41598-019-47962-3 (PMC6689018; doi:10.1038/s41598-019-47962-3)
Supplement: Supplementary file 1 — Supplementary Figures and Tables [file 41598_2019_47962_MOESM1_ESM.pdf]

Scientific Reports **Supplementary Information**

Article title: Cucumber mosaic virus-induced gene silencing in banana

Authors: Yuh Tzean[1], Ming-Chi Lee[1], Hsiao-Hsuan Jan[1,2], Yi-Shu Chiu[1], Tsui-Chin Tu[1], Bo-Han Hou[1], Ho-Ming Chen[1], Chun-Nan Chou[2], Hsin-Hung Yeh[1,2,*]

[1]Agricultural Biotechnology Research Center, Academia Sinica, No. 128, Section 2, Academia Road, Nankang District, Taipei, 11529, Taiwan.

[2]Department of Plant Pathology and Microbiology, National Taiwan University, No. 1, Section 4, Roosevelt Road, Da'an District, Taipei, 10617, Taiwan.

*Corresponding author

The following Supplementary Information is available for this article:

Fig. S1. Cucumber mosaic virus (CMV) genome and encoded proteins.

Fig. S2. Schematic representation of Cucumber mosaic virus 20 (CMV 20) infectious clones.

Fig. S3. Disease index of *Nicotiana benthamiana* inoculated with cucumber mosaic virus (CMV) inoculum.

Table S1. Reads abundance of small RNAs (21-24 bp) in banana inoculated with different constructs.

Table S2. Primers used in this study.

Fig. S1 Cucumber mosaic virus (CMV) genome and encoded proteins. RNA 1 encodes 1a protein and RNA 2 encodes 2a protein that are components of viral replicase involved in viral genome replication. RNA 2 also encodes 2b suppressor, subgenomic RNA 4A, of RNA silencing. RNA 3 encodes 3a movement protein. RNA 3 also encodes coat protein (subgenomic RNA 4). 5′ end contains 7-methyl guanosine cap; the circle at the 3′ end represent a tRNA-like secondary structure.

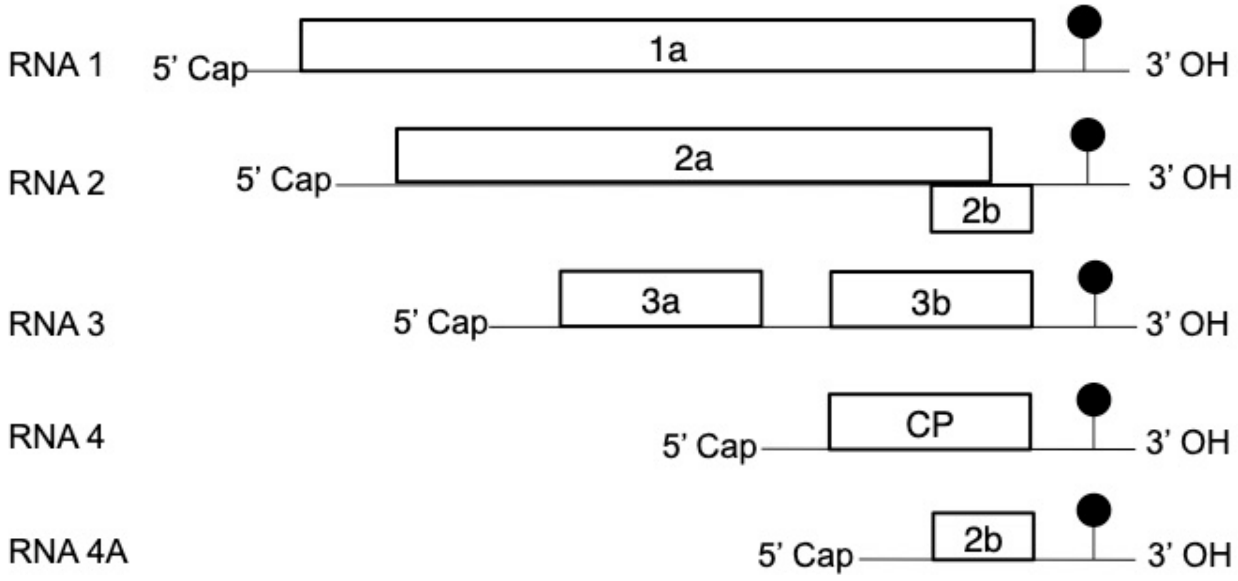

Fig. S2 Schematic representation of Cucumber mosaic virus 20 (CMV 20) infectious clones. CMV 20 infectious clones are constructed with T3 promoter immediately linked to cDNA of RNA1 (pCMV20-R1), RNA2E（pCMV20-R2E）and RNA3 (pCMV20-R3). Open rectangles represent open reading frames (ORF) encoded by CMV genomic RNA (1a, 2a, 2b, 3a, and 3b). Schematic depiction of AfeI cloning site is indicated with grey lines and triangle. Left, CMV 20 infectious construct without foreign gene insert. Right, CMV 20 with *N. benthamiana glutamate-1-semialdehyde aminotransferase* (NbGSA) in AfeI site of pCMV20-R2E.

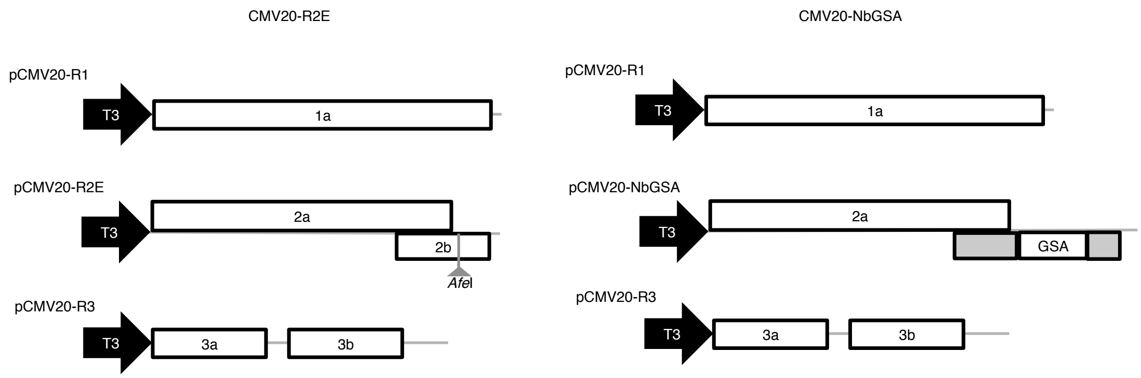

Fig. S3 Disease index of *Nicotiana benthamiana* inoculated with cucumber mosaic virus (CMV) inoculum. Plants (6-8 leaf stage) were inoculated with optimized CMV inoculum on two of the leaves. Four levels (Levels 1 to 4) of the symptoms were photographed depending on symptom development. The inoculated plants in level 1 begin to show initial leaf distortion. Level 2 indicated leaf distortion of all newly developed leaves. Level 3 indicated leaf distortion in all newly developed leaves and initial wilting of leaves. Level 4 indicated leaf distortion and severe wilting of leaves. Scale bar = 5 cm.

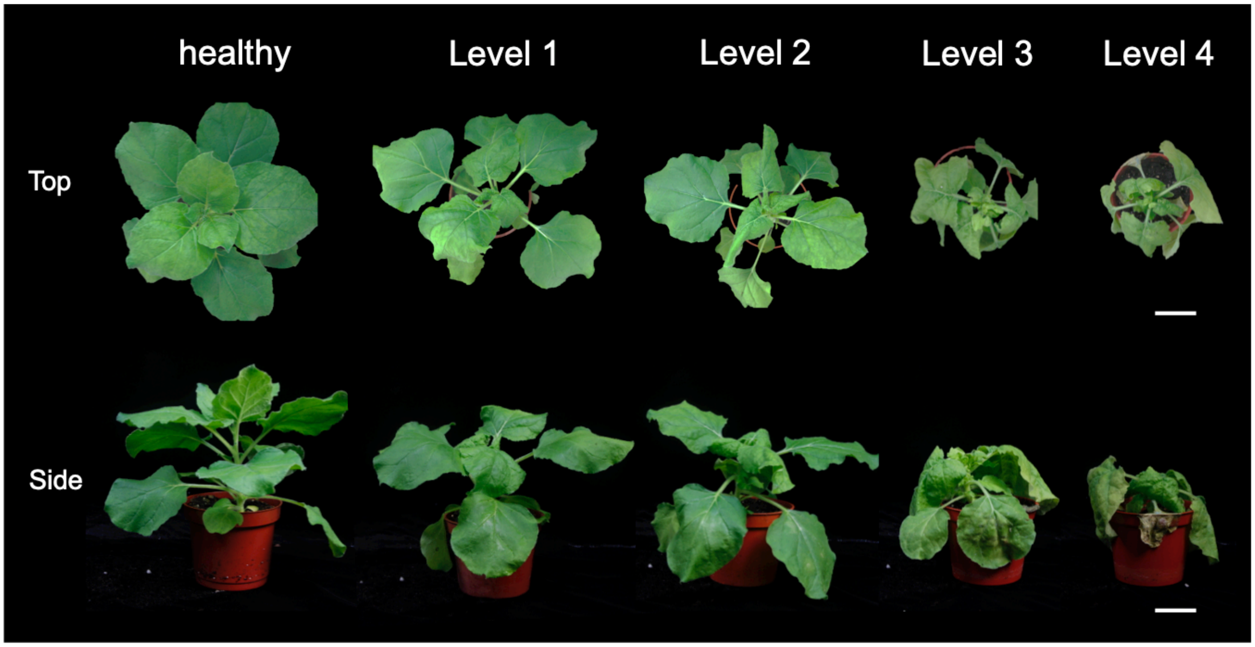

Table S1. Reads abundance of small RNAs (21-24 nt) in banana inoculated with different constructs.

Inoculum	Reads Abundance (TP10M)	
	MaGSA	MaPDS
Mock (pJL89)	74.1	29.7
CMV20-35S	174.9	78.7
CMV20-MaGSA	77990.8	63.6
CMV20-MaPDS	390.9	35506.1

Table S2. Primers used in this study.

Purpose	Primer	Primer Sequence (5'-3')
For CMV20 RNA1, 2, 3 cDNA cloning	R125	AATTAACCCTCACTAAAGTTTATTTACAAGAGCG
	CMV 3'	GCGGATCCTGGTCTCCTTT
	YR1 1646RE	TTCCCATCGGTAACAGCCTG
	YR1 1584FR	AGTGCTCATTTGACGGTGGA
	YR2 1720RE	CACCACCACTTAGTTATCGG
	YR2 1536FR	CCAGAGATGCCTTCGAGAAC
	RNA35'20-2	AATTAACCCTCACTAAGTAATCTTACCGC
For generating CMV20-R2E vectors	CMV2-2225F	GAGTTGAAATACAGGAAGTCTGGG
	CMV2-2663AfeIR	CGTAAAACAAGAGCGCTCAGACTC
	CMV2-2663AfeIF	GAGTCTGAGCGCTCTTGTTTTACG
	CMV2-2949R	GGACCGAAGTCCTTCCGAAG
For generating pJLCMV20-R1	CMV20-R1R2-F	GTTTATTTACAAGAGCGTACGGTTCAATCCCTGCC
	CMV20-R1-R	TGGTCTCCTTTAAGAGACCCCCACAAAGTG
For generating pJLCMV20-R2E	CMV20-R1R2-F	GTTTATTTACAAGAGCGTACGGTTCAATCCCTGCC
	CMV20-R2-R	TGGTCTCCTTTGAGAGACCCCCATTAAATGG
For generating pJLCMV20-R3	CMV20-R3-F	GTAATCTTACCGCTGTGTGTGCGTGTGTGTG
	CMV20-R3-R	TGGTCTCCTTTAAGGGACCCCCATTAAATGG
For VIGS of target genes on *Nicotiana benthamiana*	NbPDS-F	ATCGAGCTGAATGAGGATGG
	NbPDS-R	TGTTCTTCAGTTTTCTGTCAAACC
	NbGSA-F	GGCGGAAACAATGAAGAAAGG
	NbGSA-R	GCCAAACGCCAACCTAAATCCA
	35S-F	GAACGAGGAGCATCGTGGAAAAAG
	35S-R	CGACTAGAATAGTAAATTGTAATGTTGTTTGTTG
For VIGS of target Genes on Cavendish Bananas	MusaGSA-F	CTCCTCTGATCTTGTTAATGGTAT
	MusaGSA-R	TGAGGGAGCTAGATACACACCTTC
	MusaPDS-F	CCCGATGGAACTGTAAAGC
	MusaPDS-R	GTTCTTCAGTTTTCTGTCAAACCATA
For qRT-PCR on *Nicotiana benthamiana*	NbGSA-qF	TTATGGAGATGGTAGCACCTGCT

	NbGSA-qR	AAATCCAGCCTCAAACTGTGAT
	NbPDS-qF	GCATTTTGATTGCTTTGAACAG
	NbPDS-qR	CACAATCGGCATGCAAAGTC
	NbActin-qF	TGCCATTCTCCGTCTTGACT
	NbActin-qR	TGCAGTCTCGAGTTCCTGTT
	CMV20CP-F	TTCCTGCCTCCTCGGACTTA
	CMV20CP-R	GCTCCGTCCGCGAACATA
For qRT-PCR on Cavendish Bananas	MusaGSA-qF	CCTTGATTGAGGCCCTGAAA
	MusaGSA-qR	GGAGAACACCCATGCAAGCT
	MusaPDS2-qF	TTCTGGAAGCAACAAACTGGAA
	MusaPDS2-qR	AGTTCAGGCCTCGGGAAGTC
	MusaActin-qF	GAAGATTCTAACAGAGAGAGGATAC
	MusaActin-qR	ATCAGGTAGCTCATAGCTCTTCT
